# RETRACTION: Clinical Efficacy of Flap Transplantation Combined With Vacuum Sealing Drainage and Methylprednisolone and Cyclosporine in the Treatment of Pyoderma Gangrenosum

**DOI:** 10.1111/iwj.70559

**Published:** 2025-04-18

## Abstract

**RETRACTION:** HaoY. , HeJ. , ZhaoZ. , LiC. , and FengZ. , “Clinical Efficacy of Flap Transplantation Combined With Vacuum Sealing Drainage and Methylprednisolone and Cyclosporine in the Treatment of Pyoderma Gangrenosum,” International Wound Journal 20, no. 5 (2023): 1491–1497, 10.1111/iwj.14003.36321334 PMC10088856

The above article, published online on 02 November 2022, in Wiley Online Library (http://onlinelibrary.wiley.com/), has been retracted by agreement between the journal Editor in Chief, Professor Keith Harding; and John Wiley & Sons Ltd. Following an investigation by the publisher, all parties have concluded that this article was accepted solely on the basis of a compromised peer review process. In addition, the investigation found that the authors provided incomplete ethical approval information for the study. The editors have therefore decided to retract the article. The authors did not respond to our notice regarding the retraction.

**RETRACTION:** Y. Hao , J. He , Z. Zhao , C. Li , and Z. Feng , “Clinical Efficacy of Flap Transplantation Combined With Vacuum Sealing Drainage and Methylprednisolone and Cyclosporine in the Treatment of Pyoderma Gangrenosum,” International Wound Journal 20, no. 5 (2023): 1491–1497, 10.1111/iwj.14003.36321334 PMC10088856

The above article, published online on 02 November 2022, in Wiley Online Library (http://onlinelibrary.wiley.com/), has been retracted by agreement between the journal Editor in Chief, Professor Keith Harding; and John Wiley & Sons Ltd. Following an investigation by the publisher, all parties have concluded that this article was accepted solely on the basis of a compromised peer review process. In addition, the investigation found that the authors provided incomplete ethical approval information for the study. The editors have therefore decided to retract the article. The authors did not respond to our notice regarding the retraction.

